# Image Reconstruction Analysis for Positron Emission Tomography With Heterostructured Scintillators

**DOI:** 10.1109/trpms.2022.3208615

**Published:** 2022-09-22

**Authors:** Philipp Mohr, Nikos Efthimiou, Fiammetta Pagano, Nicolaus Kratochwil, Marco Pizzichemi, Charalampos Tsoumpas, Etiennette Auffray, Karl Ziemons

**Affiliations:** Factuly of Chemistry and Biotechnology, FH Aachen University of Applied Sciences, 52428 Jülich, Germany, and also with the Experimental Physics Department, European Organization for Nuclear Research (CERN), 1201 Geneva, Switzerland. He is now with the Department of Nuclear Medicine and Molecular Imaging, University of Groningen, University Medical Center Groningen, 9713 GZ Groningen, The Netherlands; Department Radiology, Perelman School of Medicine, University of Pennsylvania, Philadelphia, PA 19104 USA; Physics Department, University of Milano-Bicocca, 20126 Milan, Italy, and also with the Experimental Physics Department, European Organization for Nuclear Research (CERN), 1201 Geneva, Switzerland; Experimental Physics Department, European Organization for Nuclear Research (CERN), 1211 Geneva, Switzerland; Physics Department, University of Milano-Bicocca, 20126 Milan, Italy, and also with the Experimental Physics Department, European Organization for Nuclear Research (CERN), 1201 Geneva, Switzerland; Department of Nuclear Medicine and Molecular Imaging, University of Groningen, University Medical Center Groningen, 9713 GZ Groningen, The Netherlands, and also with the Biomedical Imaging Science Department, Leeds Institute of Cardiovascular and Metabolic Medicine, University of Leeds, LS2 9JT Leeds, U.K.; Experimental Physics Department, European Organization for Nuclear Research (CERN), 1211 Geneva, Switzerland; Faculty of Biomedical Engineering and Technomathematics, FH Aachen University of Applied Sciences, 52428 Jülich, Germany

**Keywords:** Heterostructure, image reconstruction, metascintillator, multiple TOF kernels, TOF positron emission tomography (PET)

## Abstract

The concept of *structure engineering* has been proposed for exploring the next generation of radiation detectors with improved performance. A TOF-PET geometry with heterostructured scintillators with a pixel size of 3.0 × 3.1 × 15 mm^3^ was simulated using Monte Carlo. The heterostructures consisted of alternating layers of BGO as a dense material with high stopping power and plastic (EJ232) as a fast light emitter. The detector time resolution was calculated as a function of the deposited and shared energy in both materials on an event-by-event basis. While sensitivity was reduced to 32% for 100-*μ*m thick plastic layers and 52% for 50 *μ*m, the coincidence time resolution (CTR) distribution improved to 204 ± 49 and 220 ± 41 ps, respectively, compared to 276 ps that we considered for bulk BGO. The complex distribution of timing resolutions was accounted for in the reconstruction. We divided the events into three groups based on their CTR and modeled them with different Gaussian TOF kernels. On an NEMA IQ phantom, the heterostructures had better contrast recovery in early iterations. On the other hand, BGO achieved a better contrast-to-noise ratio (CNR) after the 15th iteration due to the higher sensitivity. The developed simulation and reconstruction methods constitute new tools for evaluating different detector designs with complex time responses.

## I. Introduction

Positron Emission Tomography (PET) is a highly sensitive imaging modality in nuclear medicine that reveals the metabolic or biochemical functions of tissues and organs. Positron emission tomography (PET) can image many cellular pathways of receptors, providing global and regional assessments of diseases. The scanner detects pairs of 511-keV gamma rays emitted from electron-positron annihilations propagating along a line of response (LOR) [1].

Arguably, the two driving factors for the sensitivity of PET systems are the scanner’s geometry and the stopping power of the detector’s scintillating material. Inorganic scintillators with high density and effective atomic numbers like Cerium doped Lutetium Oxyorthosilicate (LSO/LYSO) or bismuth germanate (BGO) are commonly used due to their high gamma-ray stopping power [2], among other factors.

Another critical requirement for modern PET scanners is to exhibit excellent coincidence time resolution (CTR). By measuring with high accuracy the detection time difference between the two gamma-rays, we can restrict the likelihood of the annihilation’s position in the LOR; this is known as TOF-PET [3], [4]. It has been proven that improving the CTR increases the signal-to-noise ratio (SNR) gain in the reconstructed images [5]. It even has the potential to overcome the limitations induced by the physical size of detectors on spatial resolution [6]. TOF offers significantly better image quality (IQ) which can be translated to shorter acquisition times and/or lower injected doses—a tradeoff requiring careful consideration in new PET protocols [4], [7], [8], [9], [10], [11]. Nowadays, the CTR of commercially available PET scanners is in the range of 200 to 300 ps [12], [13].

The major advantage of inorganic scintillators is their high stopping power for 511-keV gamma-rays. On the other hand, organic scintillators have better CTR for PET [14], [15], but lower density and effective atomic number; thus, their low stopping power presents a severe drawback for their wide adoption.

Heterostructured scintillators attempt to alleviate the natural limitations of the above scintillators by combining them in one detector, aiming for only the advantageous properties. These next-generation scintillation detectors are based on the concept of structure engineering [16], [17], [18]. The basic idea is that the dense inorganic scintillator stops the gamma-ray. At the same time, the recoil electron deposits some of its energy in the fast organic material, resulting in better timing resolution. In literature, the term metascintillator has been recently used to describe heterostructured scintillators [19], picking up the concept of metamaterials in material science.

An experimental proof-of-concept for a heterostructured scintillator was presented in [20] using 3.8 × 3 .8 × 3 mm^3^ pixels of 200-*μ*m thick layers of alternating BGO or LYSO with a fast plastic scintillator (BC422). The authors identified different types of events with standard CTR of the bulk material or improved CTR due to energy deposition in the fast emitter. In a follow-up work [21] a similar design for longer (3 × 3 × 15 mm^3^) crystals and different layer thicknesses was investigated. Further results on LYSO and BGO-based heterostructures were reported in [22]. Both works note that the heterostructure layers should be smaller than the recoil range of the electrons to allow energy sharing between the two materials. In BGO, the average range of the primary electrons generated by 511 keV gamma-rays is below 100 *μ*m, whereas for LYSO it is slightly above 100 *μ*m [21], [23].

Recently, based on Monte Carlo simulations of different heterostructure configurations, general design guidelines were proposed [24]. The authors stress the importance of maximizing the fraction of fully absorbed events, possibly by increasing the detectors’ length and facilitating energy sharing by increasing the fast material’s thickness. The authors discussed the various contradictions they faced, which we will also be discussing in later paragraphs.

In this article, we investigated the potential impact on PET imaging and IQ of combining a dense, inorganic scintillator with a fast, organic one. For each registered event, we considered the time resolution as a function of the energy deposition in each material, especially in the fast scintillator. The above led to an interesting tradeoff which we sought to investigate.

On the one hand, a larger volume fraction of the organic material in the heterostructured scintillator has a positive impact on the CTR, while on the other hand, it also decreases the stopping power. Predicting the impact of the volume fraction and the sampling frequency (number of layers) is not trivial. To that end, we used Monte Carlo simulations for two examples of BGO/plastic heterostructured scintillators. We decided to investigate BGO as the dense component of the heterostructure because of its high stopping power, cost-effectiveness, and promising results for time resolution due to Cherenkov photons’ contribution [25]. For the fast emitter, we chose the plastic scintillator EJ232 (the Eljen Technology) [26] as it combines a fast signal (rise time below 50 ps, effective decay time [27] about 1.5 ns) and a high light output (8000–10 000 ph/MeV). The properties of EJ232 are very similar to BC422 (Saint-Gobain), which was used by [20]. In the image reconstruction, we exploited the fraction of events with a faster CTR by applying different timing kernels [28]. We compare the performance of a heterostructure-based scanner to one with bulk BGO detectors in terms of count rates and the quality of the reconstructed images in terms of contrast recovery coefficient (CRC) and contrast-to-noise ratio (CNR) using the NEMA IQ phantom. For BGO, we considered two-time resolutions, one from our laboratory measurements that include the exploitation of Cherenkov photons and a larger value to account for expected deterioration when approaching system levels.

## II. Materials and Methods

### A. Time Resolution Function for Heterostructured Scintillators

In this work, we implemented a function to calculate the time resolution of each event in the Monte Carlo simulation based on the deposited energy. This analytic model accounts for the energy sharing-dependent time resolution of the heterostructured scintillators.

Typically, in PET, the time resolution is assumed to be broadly the same (mainly affected by the rate of single events) for the two detectors [29] and the relationship between detector time resolution (DTR) and CTR can be given as $\text{CTR}=\sqrt{2}\cdot \text{DTR}$. However, for the general case where the two detectors may have different time resolutions, the CTR should be expressed as $\text{CTR}=\sqrt{{\text{DTR}}_{1}^{2}+{\text{DTR}}_{2}^{2}}$, where DTR_1_ and DTR_2_ are the time resolutions of the two detectors involved in the coincidence. In heterostructures, the DTR is a function of energy deposition in both materials, which is a statistical process; therefore, in general, the time response of the two detectors is different.

The model of the energy-dependent DTR [DTR*(E)*] can be given by Vinogradov’s equation [30]

(1) $$\text{DTR}(E)=\frac{1}{\sqrt{\text{IPTD}(E)}}$$

with

(2) $$\text{IPTD}(E)=\frac{\text{ILY}(E)}{\tau_{\text{deff }}\left(1.57\tau_{r}+1.13\sigma_{\text{SPTR}+\text{PTS}}\right)}$$

where *τ*_deff_ is the effective decay time, *τ*_*r*_ the scintillation rise time, ILY the intrinsic light yield, and *σ*_SPTR+PTS_ combines the contribution due to the single photon time resolution (SPTR) of the silicon photomultiplier (SiPM) and the photon transfer time spread (PTS).

In the above empirical equation, the DTR*(E)* is proportional to the inverse of the square root of the initial photon time density (IPTD), which is, in turn, proportional to the energy-dependent ILY. In [27] it was shown that the equation provides a reasonable estimate of the measured time resolution of various scintillators.

The novelty in heterostructured scintillators is that the time resolution is a function of the energy deposition in two materials. The idea is that the IPTDs of the individual materials can be added to determine the combined time resolution

(3) $$\text{DTR}\left(E_{\text{Pl}},E_{\text{BGO}}\right)=\frac{1}{\sqrt{{\text{IPTD}}_{\text{Pl}}\left(E_{\text{Pl}}\right)+{\text{IPTD}}_{\text{BGO}}\left(E_{\text{BGO}}\right)}}$$

where IPTD_Pl_(*E*_Pl_) is the IPTD caused by the plastic scintillator as a function of the energy deposited in the plastic. Similarly, IPTD_BGO_(*E*_BGO_) is the IPTD caused by the energy deposited in BGO. Since the individual IPTDs are proportional to the energy deposited in the specific material, we can calculate them as follows:

(4) $${\text{IPTD}}_{\text{Pl}}\left(E_{\text{Pl}}\right)=\frac{E_{\text{Pl}}/340}{{\text{DTR}}_{Pl@340\text{keV}}^{2}}$$

(5) $${\text{IPTD}}_{\text{BGO}}\left(E_{\text{BGO}}\right)=\frac{E_{\text{BGO}}/511}{{\text{DTR}}_{\text{BGO}@511\text{keV}}^{2}}$$

where the time resolutions we used were measured with the individual bulk materials and normalized to the reference energies of the Compton edge (340 keV) for the plastic scintillator and the photopeak (511 keV) for BGO. The time resolutions of the individual bulk materials were measured for a geometry of 3 × 3 × 15 mm^3^ with the same setup as in [27]. The scintillators were wrapped in Teflon and coupled to the photodetector using Meltmount.

With 3 × 3 × 15 mm^3^ scintillator pixels, we measured CTRs of 271 ps for BGO and 94 ps for the plastic scintillator EJ232 (Eljen Technology) at 511 and 340 keV, respectively. Based on the above measurements, we calculated the DTR_*Pl*@340keV_ and DTR_BGO@511keV_.

It has to be noted that the model we used here is a simple approximation and does not consider effects due to the thin plate-shaped structure, such as different light transport and stronger depth of interaction (DOI) bias. Indeed, we have treated the DOI impact similar to the DOI of typical 15-mm crystals, which we will describe in Section II-C.

To summarize, our model is a simple method to calculate the CTR on an event-by-event basis and study the influence of the resulting CTR distribution on the reconstructed image.

Furthermore, we acknowledge that the input values (DTR_*Pl*@340keV_ and DTR_BGO@511keV_) come from laboratory measurements with optimized conditions regarding readout electronics, data acquisition, and analysis on single pixels. For this reason, we simulated two time resolutions for the BGO model. One from laboratory measurements that allow us to exploit the signal of the fast Cherenkov photons [25]. And a second, larger value typical for standard PET, measuring only scintillation photons with a deterioration, as seen in a whole PET system [22].

### B. Monte Carlo Simulations

Simulations provide insight into the fractions of energy deposited in plastic and BGO layers enabling an understanding of energy sharing between the two materials and the resulting DTR. We performed Monte Carlo simulations using a modified GATE toolkit (v8.2) [31]. We implemented the DTR*(E*_Pl_*, E*_BGO_) function in the readout module (GateReadout class). In this class, we separate the energies deposited in the BGO and plastic layers and use them to generate a final output pulse with a DTR as described in (3), that varies on an event-by-event basis.

We simulated a cylindrical PET geometry with a diameter of 701.0 mm and an axial length of 99.0 mm. The system consisted of 33 detector rings each with 710 detectors with size 3.0 × 3.1 × 15.0 mm^3^. This arrangement led to a PET geometry as close to a cylinder as possible, avoiding any gaps between the detectors and layers. We kept the axial length below 10 cm to restrain the computational effort required for simulation and reconstruction at reasonable levels.

**Fig. 1.**
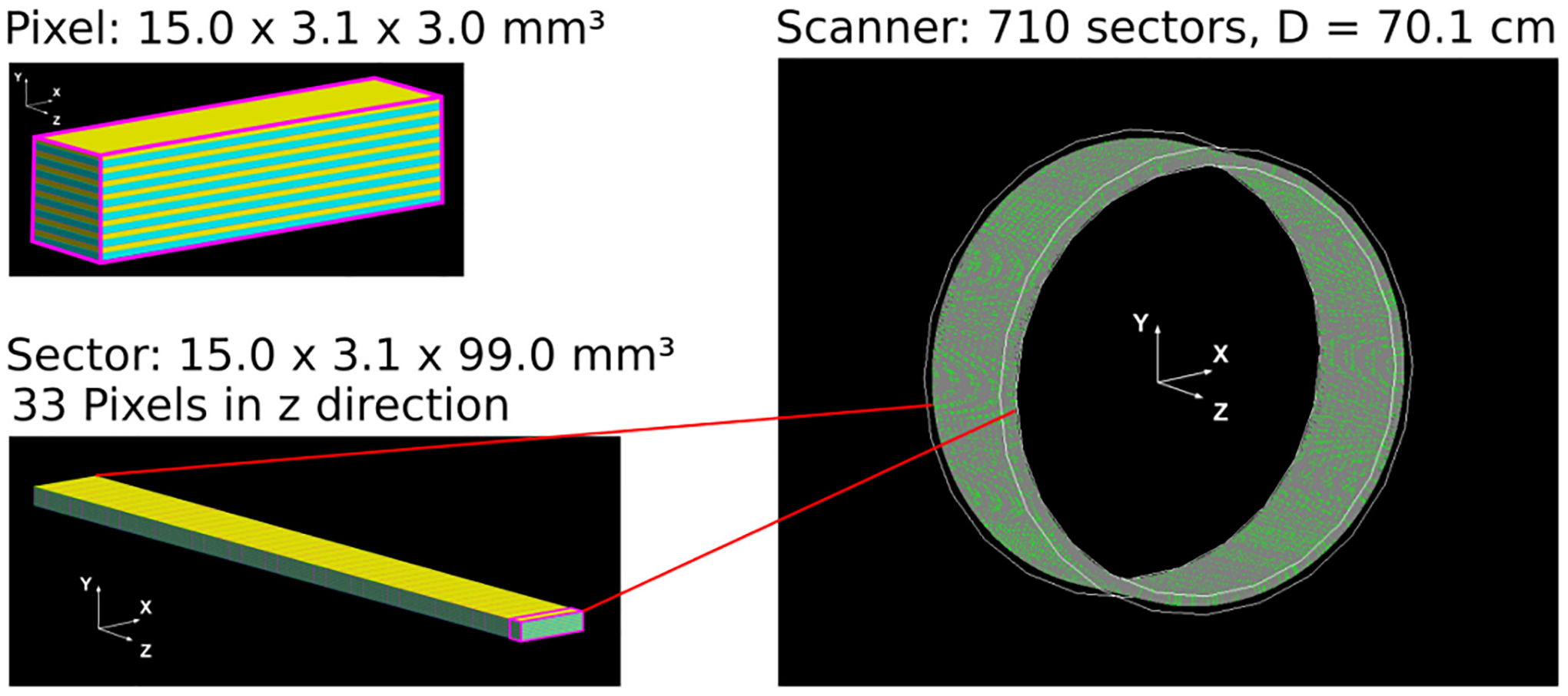
(Top left) a single BGO/plastic heterostructured scintillator is demonstrated. (Bottom left) the single heterostructure was repeated in the axial direction to create the scanner’s modules. (Right) the entire cylindrical scanner is shown after repeating the sector with the GATE ring repeater.

Each heterostructured scintillator consisted of a stack of alternating BGO and plastic layers along the transaxial direction (Fig. 1). In this study, we investigated two heterostructure models; one with, equal, 100 *μ*m BGO and plastic layers (31 layers in total) and one with 100 *μ*m BGO and 50 *μ*m plastic layers (41 in total). For simplicity, throughout this article, these two geometries will be referred to as Hetero-Pl-100 and Hetero-Pl-50.

To reduce the effect of energy escaping the detector, all heterostructures started and ended with a BGO layer. We chose the thickness of 100 *μ*m for BGO based on the range of recoil electrons in BGO [23] and to be close to what was used experimentally in [21]. By choosing 100 and 50 *μ*m plastic layers, the sensitivity of LYSO is between the sensitivity of these two configurations (as shown later in Fig. 5). Our reference model was based on bulk BGO crystals of 3.0 × 3.1 × 15 mm^3^ and was simulated with the same energy-dependent DTR model.

We set the Geant4 physics list to emstandard_opt3 with an additional reduction of the production cuts from the default 1 mm to 5 *μ*m in the detector volumes to allow a more accurate simulation of the energy distribution between the thin layers of 50 and 100 *μ*m.

We must note that the simulations recorded the energy deposition from each gamma-ray in the two materials, and we did not simulate optical photons, surfaces, or photosensors. The deposited energies were input to the DTR function based on experimental measurements.

The coincidence window was set to 4 ns and the GATE parameter minSectorDifference to 88 [32]. In previous work on TOF PET reconstruction applied on the Cherenkov radiation in BGO [28], an energy resolution of 19% was considered, whereas, for the heterostructured scintillators, worse resolution can be expected due to the layered structure. However, here, to isolate the effects of CTR on the reconstruction of TOF PET image reconstruction, the same energy resolution 20% and an energy window of 400–650 keV were used in all models.

We simulated the geometry of a typical NEMA IQ phantom [33] with four hot spheres (diameters 10, 13, 17, and 22 mm). The background activity was 42.9 MBq, and the hot sphere ratio was 4:1. The simulated acquisitions’ duration was 2000 s.

### C. Depth of Interaction Contribution in the Simulations

While in (2) the PTS as additional jitter similar to the SPTR was considered and becomes less important for high ILY, this is not the case for the DOI contribution for long crystals [32]. This is caused by the natural uncertainty in the gamma ray’s absorption point inside the crystal; thus, its contribution cannot be ignored during the simulation. To keep the simple analytic model, the input DTR values (DTR_*Pl*@340keV_ and DTR_BGO@511keV_) were precorrected for the DOI contribution to get at the output of the desired CTR.

To roughly estimate the CTR without DOI (denoted as CTR′), we simulated a point source at the center of the field of view (FOV). We fitted a Gaussian function on the time differences of the timestamps of the photoelectric absorption of the two in-coincidence events. We found an FWHM of 51 ps and subtracted it from the target CTR in quadrature. The simulated DOI value of 51 ps is very close to the empirical value of 50 ps, which corresponds to the length of the crystal divided by the speed of light, as shown by the observation in [34] this empirical formula works for LSO crystals.

In Table I, we summarize the simulated time resolutions. In the first column, we specify the target CTR we aim to achieve at the end of the simulation. The second column shows the CTR after correction for DOI; this is an intermediate value we use to calculate the DTR. Finally, in the third column, we show the DTR_*Pl*@340keV_ and DTR_BGO@511keV_ as per (4) and (5) that we used to obtain the targeted CTRs. We simulated three different input time resolutions for plastic (DTR_*Pl*@340keV_) and two different for BGO (DTR_BGO@511keV_).

The plastic resolutions include the experimental value of Pl-*τ* 1 = 94 ps, and two faster resolutions: 1) Pl-τ 2 = 75 ps and 2) Pl-*τ* 3 = 51 ps. With the faster resolutions, we aim to investigate the influence of having a material with higher IPTD and a similar density of plastic to account for the next detector generation replacing plastic with nanocomposites [35], [36].

The time resolutions for BGO were BGO-*τ* 1 = 271 ps (laboratory value) and BGO-*τ* 2 = 500 ps. The latter resembles a poorer BGO resolution based on literature values for BGO [22], [37] with older or less optimized setups. At the same time, the poorer BGO resolution accounts for expected deterioration when approaching the level of a whole PET system.

### D. Image Reconstruction

We reconstructed the data with the TOF LM-MLEM, as implemented in the open-source image reconstruction toolkit software for tomographic image reconstruction (STIR) [34], [38], [39]. We excluded random and scattered events identified by the GATE simulation, thus only reconstructing trues. The data were sorted in 355 views ×351 tangential positions over 33 segments. The timing differences were discretized in 1-ps bins, with an integration size of 0.149 mm. An odd number of TOF bins was used to get a centered TOF bin.

The voxel size of the reconstructed images was 2 × 2 × 1.5 mm^3^ on 160 × 160 × 65 grid. All configurations were reconstructed with 100 iterations.

We calculated the attenuation correction analytically with the linear attenuation values found in NIST [40]. For the normalization calculation, we simulated a cylindrical back-to-back source with a diameter of 660 mm, covering the entire FOV without any attenuation. The simulations collected over 10^9^ events for each detector configuration.

**Fig. 2.**
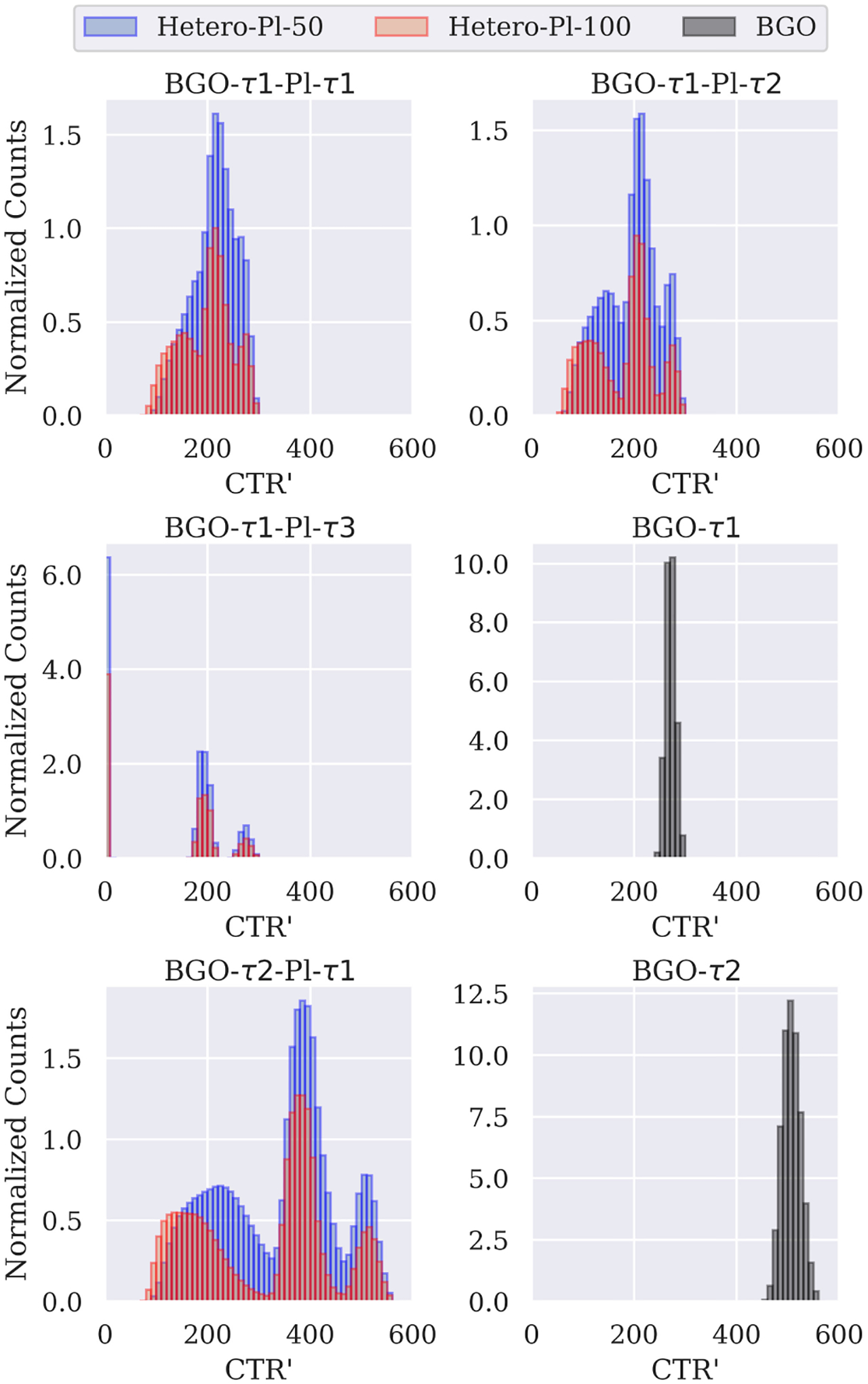
CTR′ distributions for all simulated data sets with input resolutions as given in Table I. Three peaks can be clearly distinguished for the heterostructure configurations. For visualization purposes, the counts were normalized to the amplitude in the second group of the BGO-*τ* 1-Pl-*τ* 1 configuration.

In Fig. 2 we show the CTR′ distributions (without DOI contribution) for all simulated data sets. As one can see, the BGO events are clustered around a single peak. On the other hand, the heterostructures have three peaks in their distributions. Each peak corresponds to different combinations between detectors 1 and 2. The first peak contains shared events on both detectors (fast–fast). In the second peak, energy sharing took place in one of the two detectors (fast–slow), and in the third peak, we had BGO-only interactions (slow–slow). The shape of the peaks depends on the input time resolutions, and we see that they are better separated with faster plastic (Pl-*τ* 3 compared to Pl-*τ* 1) and slower BGO (BGO-*τ* 2 compared to BGO-*τ* 1).

To simplify the reconstruction model, we divided the CTR values into three groups (*g*) modeled with different Gaussian kernels. The boundaries of these groups were chosen as a compromise based on the local minima visible in the CTR distributions in Fig. 2. For the BGO-*τ* 1-Pl-*τ* 1 and BGO-*τ* 1-Pl-*τ* 2 cases, we applied constant thresholds at 175 and 250 ps. For BGO-*τ* 1-Pl-*τ* 3, we adjusted the thresholds to ensure that all events of the three groups were separated. For BGO-*τ* 2-Pl-*τ* 1 the constant thresholds were 320 and 460 ps. For each group (*g*), the TOF kernel (*f*_*g*_) width in the reconstruction was the unweighted arithmetic mean ${\bar{\text{CTR}\prime }}_{g}$ of the applied resolutions inside the boundaries. However, as discussed in Section II-C, to avoid underestimating the width of the TOF kernel, we added in quadrature the DOI contribution

(6) $$f_{g}=\sqrt{{\bar{\text{CTR}\prime }}_{g}^{2}+{\text{DOI}}^{2}}.$$

We give all ${\bar{\text{CTR}\prime }}_{g}$ and *f*_*g*_ values in Table II, as well as the proportion of each group as a percentage of the total number of events.

### E. Figures of Merit

This study used the CRC and CNR. For the hot spheres, the CRC is calculated as follows:

(7) $${\text{CRC}}_{r}=\frac{\left(\frac{\mu_{H,d}}{\mu_{B,d}}-1\right)}{\alpha -1}\cdot 100\%$$

where *μ*_*H*_ is the mean value in a spherical region of interest (ROI) with diameter *d*, that of the respective sphere (*r*), *μ*_*B*_ is the mean value in the background taken using 24 circular ROIs in the two central slices, and *α* is the actual activity ratio, which is 4 in this case.

The CNR is given by

(8) $$\text{CNR}=\frac{\mu_{H,d}-\mu_{B,d}}{\sqrt{\sigma_{H,d}^{2}+\sigma_{B,d}^{2}}}$$

where *σ*_*H*_ and *σ*_*B*_ are the standard deviations in the hot spheres and the background, respectively.

## III. Results

### A. Time Resolution as Function of Energy Sharing

**Fig. 3.**
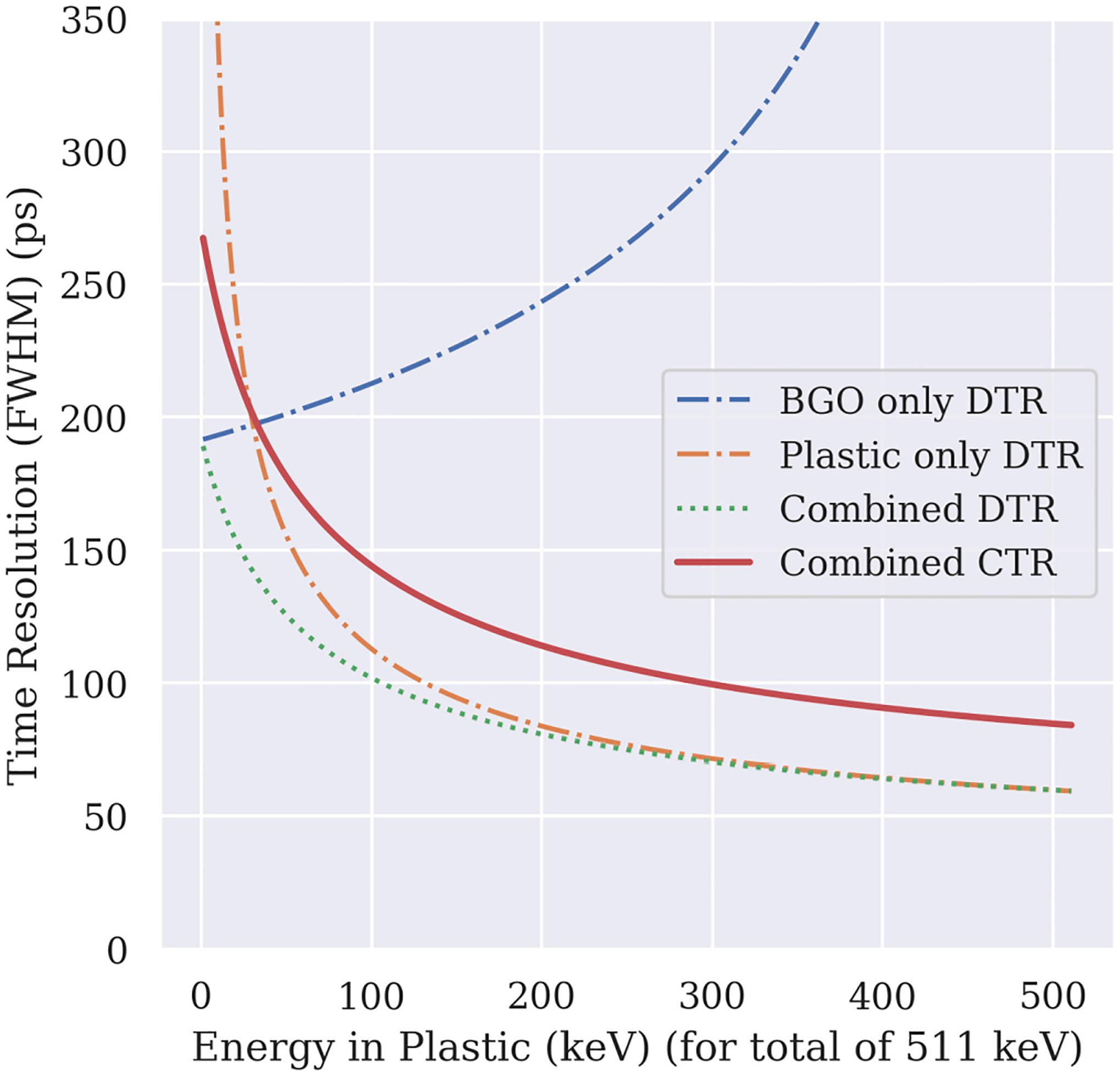
Analytically calculated time resolution as a function of the energy in plastic for a total of 511 keV with CTR input values of 271 ps for BGO and 94 ps for plastic. Shown are the individual DTRs of both materials and the combined resolution expressed as DTR and CTR, assuming the same energy was deposited in both detector pairs $\left(\text{CTR}=\sqrt{2}\cdot \text{DTR}\right)$. With increasing energy in plastic, the faster plastic scintillator dominates the time resolution.

In Fig. 3, we show the response of DTR*(E*_BGO_*, E*_Pl_) as a function of *E*_Pl_ from the initial energy of 511 keV for an input time resolution of 271 ps for BGO and 94 ps for plastic. We also visualize the individual contributions of the energies deposited in each material to the combined CTR by setting either *E*_BGO_ = 0 or *E*_Pl_ = 0 in (3). By doing so, “BGO only” and “Plastic only” show the time resolution based only on *E*_BGO_ or *E*_Pl_.

We see in the curves of Fig. 3 that the DTR of BGO layers gets progressively worse as more energy is deposited in the plastic. In contrast, the DTR from solely the *E*_Pl_ (“plastic only”) improves monotonically. As demonstrated by the curves of “combined DTR” and “combined CTR,” the time resolution improves with more energy deposited in the plastic layers.

Finally, when the energy deposited in the plastic exceeds 300 keV, the combined DTR is nearly the same as the “plastic only” case, which indicates that the fast photons drive the CTR values.

**Fig. 4.**
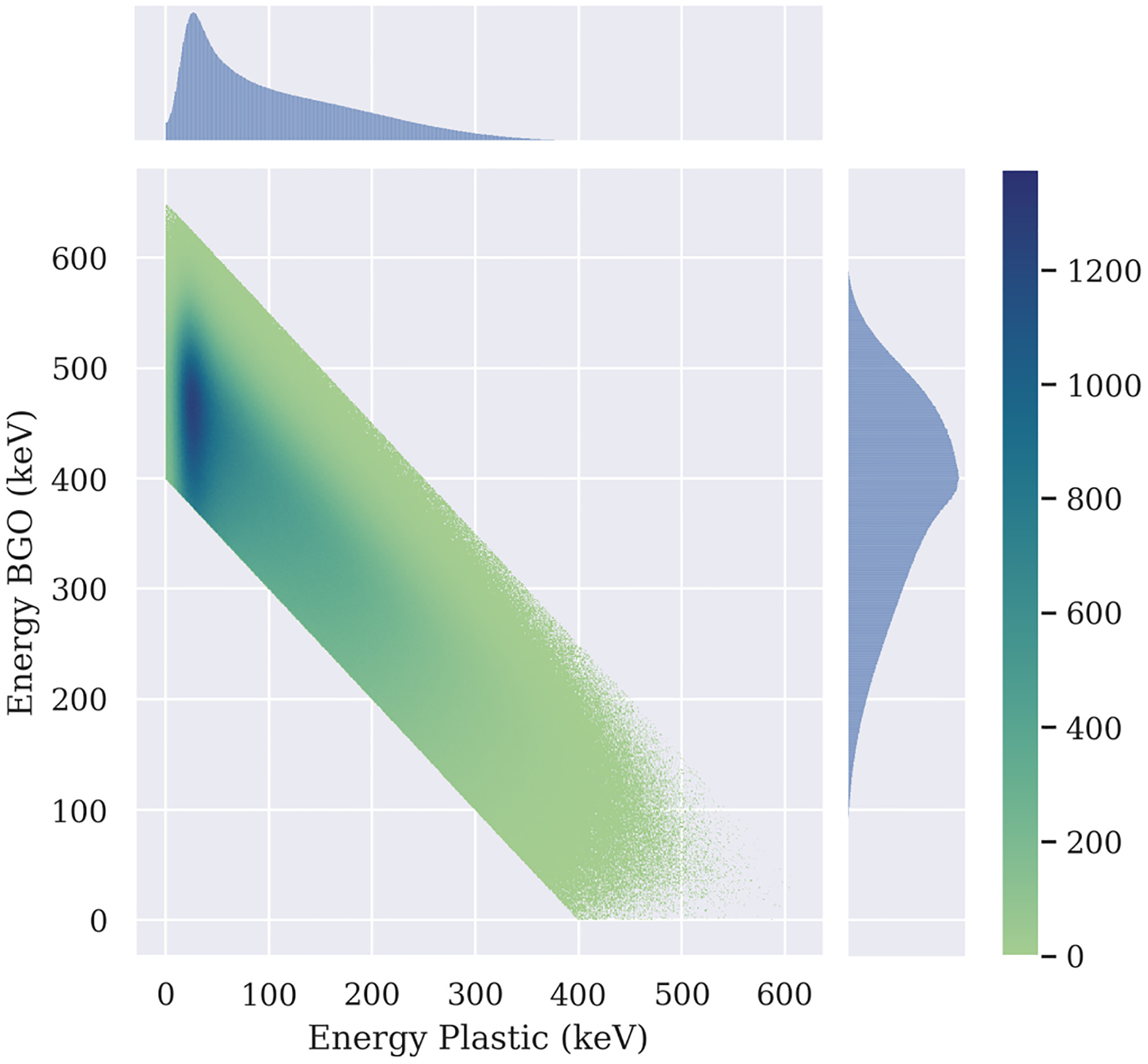
Energy distribution between both materials of Hetero-Pl-100 geometry. Only shared events are shown. Color scale (*z*-axis) shows counts of events with specific energy distribution in corresponding bins.

In Fig. 4 the energy distribution of the shared events (*E*_Pl_ > 0 keV) of Hetero-Pl-100 geometry, is shown, based on an acquisition of 2000 s resulting in 49.8 × 10^6^ prompt coincidences. In this case, the shared events account for 63.5% of the total. The rest deposited their energy only in BGO. We can observe that most shared events deposited only a low fraction of their energy in plastic. We summarize information about the proportion of shared events with *E*_Pl_ > 0 keV and *E*_Pl_ > 50 keV for the two configurations Hetero-Pl-100 and Hetero-Pl-50 in Table III.

For the two configurations investigated in this work, the proportion of shared events remains about the same (driven by the 100 *μ*m BGO thickness). Still, the proportion of events with more than 50 keV deposited in plastic is higher for the thicker plastic layers. For instance, the average deposited energy and the standard deviation is 108.4 ± 83.4 and 65.7 ± 60.1 keV for Hetero-Pl-100 and Hetero-Pl-50, respectively.

### B. Time Resolution Over Sensitivity

**Fig. 5.**
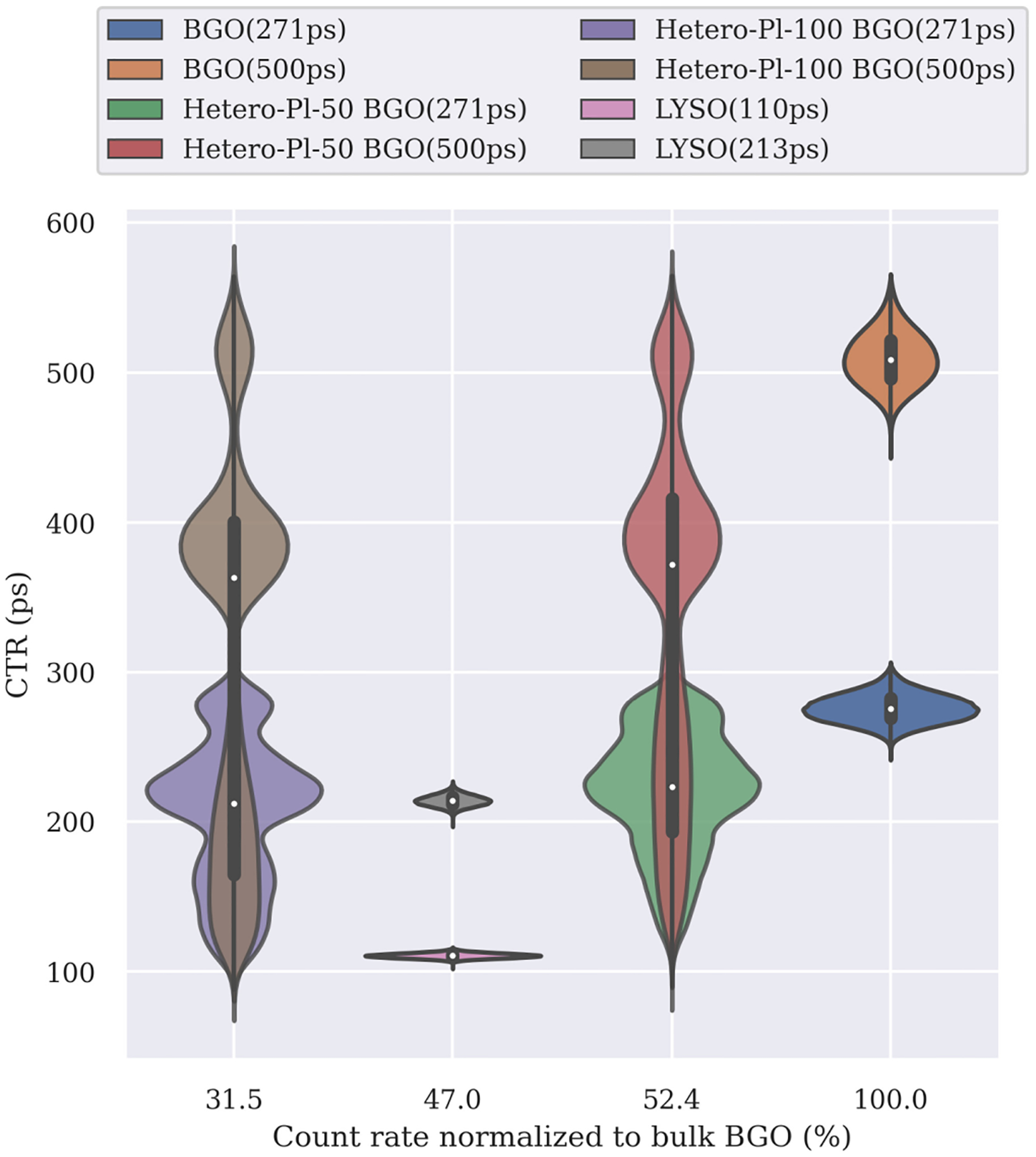
CTR versus true coincidence rate normalized to bulk BGO for different scanner configurations. 400–650 keV energy window and 20% resolution for BGO and Heterostructure, 450–650 keV energy window and 11% resolution for LYSO. This study simulated an equivalent CTR of 271 or 500 ps for BGO and 94 ps for plastic. For LYSO, the value 213 ps represents the Siemens Biograph Vision PET scanner and 110 ps was approximated for LYSO under laboratory conditions based on [27]. The violin plots show the distribution of CTR values for each simulated scanner configuration.

In Fig. 5, we show violin plots of the CTR distributions for the Heterostructure configurations with the experimental value of Pl-*τ* 1 = 94 ps and the two bulk scintillators BGO and LYSO. On the *x*-axis, we show the drop in the count rate as a percentage of bulk BGO.

As it can be seen, the use of thicker plastic layers leads to improved CTR; however, it also results in a noticeable reduction in the count rate down to 31.5% of BGO’s. While with thinner plastic layers, the value is 52.4%. Using 271 ps as input time resolution for BGO, the CTR distributions show mean and standard deviation of 204 ± 49, 220 ± 41, 276 ± 9 ps for Hetero-Pl-100, Hetero-Pl-50, and bulk BGO (271 ps), respectively.

For BGO with 500 ps input time resolution, the corresponding values are 317 ± 127, 344 ± 112, 509 ± 18 ps for Hetero-Pl-100, Hetero-Pl-50, and bulk BGO (500 ps), respectively.

We should note that the CTR distributions of heterostructures show a spread of values between approximately 100 and 300 ps for BGO (271 ps) and 100 and 600 ps for BGO (500 ps). Considerably wider than bulk materials and with multiple peaks.

For comparison, in Fig. 5 we included simulations of LYSO detectors with input CTRs of 213 (Siemens Biograph Vision [12]) and 110 ps (laboratory conditions [27]). We see that the count rate of LYSO falls in-between the two heterostructure configurations with a coincidence rate of 47.0% that of BGO and resulted in a CTR of 214 ± 4 and 111 ± 2 ps for an energy threshold of 450 keV and 11% energy resolution.

Moreover, Fig. 5 suggests that Hetero-Pl-50, especially combined with a fast BGO, can be very competitive with LYSO in terms of CTR.

### C. Image Quality

We summarize basic statistics on the NEMA IQ [33] simulated data sets used in reconstruction in Table IV. The measured drop in true counts was 32% and 52% for Hetero-Pl-100 and Hetero-Pl-50, respectively.

**Fig. 6.**
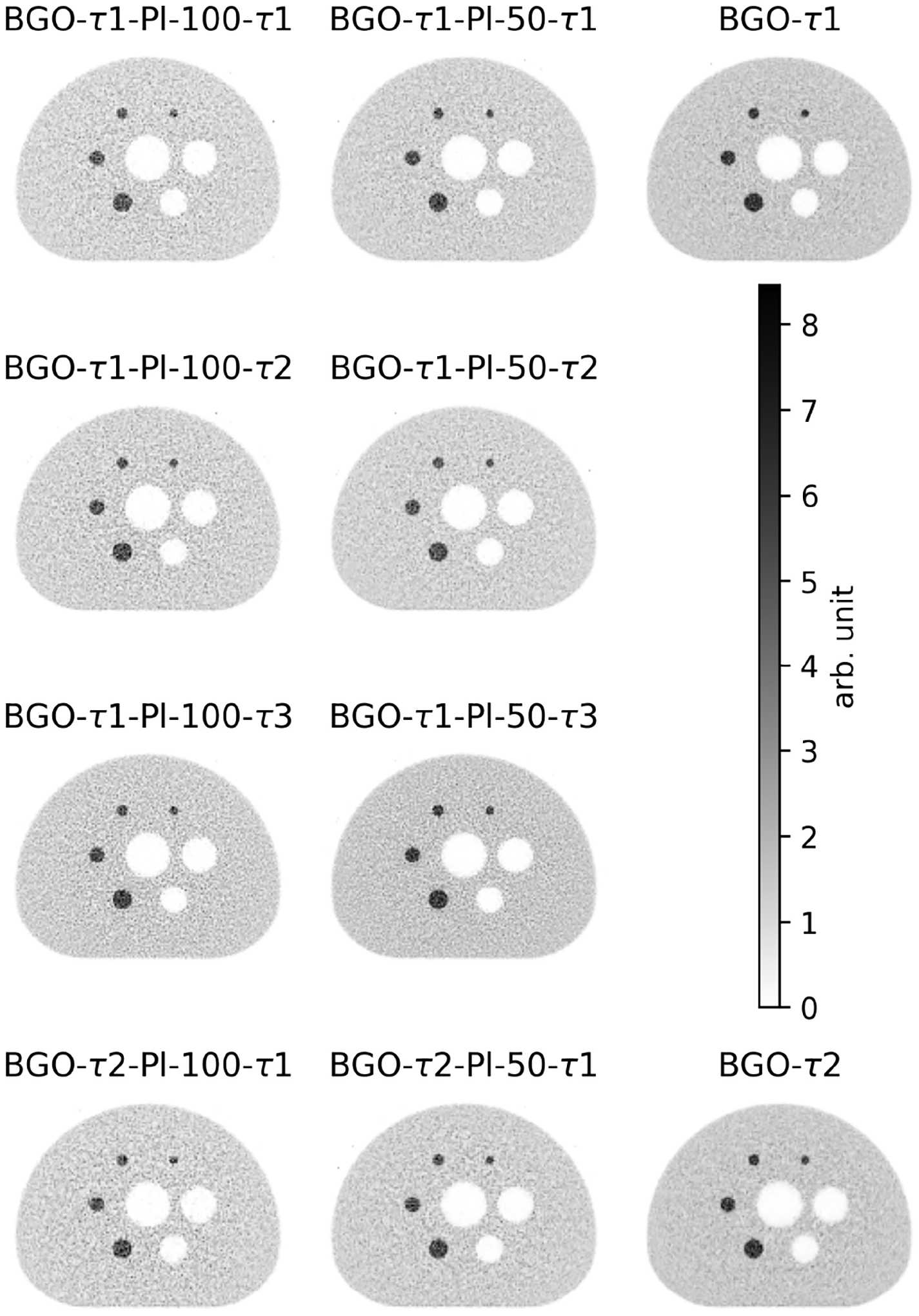
Reconstructed images at the 60th iteration for three scanner configurations Hetero-Pl-100, Hetero-Pl-50, and BGO, for two different BGO time resolutions: BGO-*τ* 1 = 271 ps and BGO-*τ* 2 = 500 ps and for three different plastic time resolutions: Pl-*τ* 1 = 94 ps, Pl-*τ* 2 = 75 ps, and Pl-*τ* 3 = 51 ps.

In Fig. 6, we show the images obtained at the 60th iteration for all simulated scanner models. Due to the drop in stopping power and disproportional improvement in the time resolution, we can see in the images higher noise when thicker plastic layers are used.

The background variability (BV) in the images at the 60th iteration, given by the standard deviation divided by the mean of the 24 background ROIs with *d* = 22 mm, is 2.0%, 2.5%, and 3.6% for the BGO-*τ* 1, BGO-*τ* 1-Pl-50-*τ* 1, and BGO-*τ* 1-Pl-100-*τ* 1 images, respectively, using an input time resolution of 271 ps for BGO. The corresponding values for an input time resolution of 500 ps for BGO are 1.4%, 2.2%, and 3.5%. However, it is not possible to directly compare the BV between the three images as they have been reconstructed with different time resolutions; thus, MLEM has converged at different rates. For instance, we see that with 75-ps plastic time resolution (Pl-*τ* 2), the BV is 2.7% and 3.6% for BGO-*τ* 1-Pl-50-*τ* 2 and BGO-*τ* 1-Pl-100-*τ* 2, which shows that convergence can further speed up using faster plastic with thinner layers, while there may be no additional benefit for the case of Pl-100. The above suggests that even if the input time resolution of the material improves, the perceived TOF effect still depends on the average deposited energy in the plastic.

**Fig. 7.**
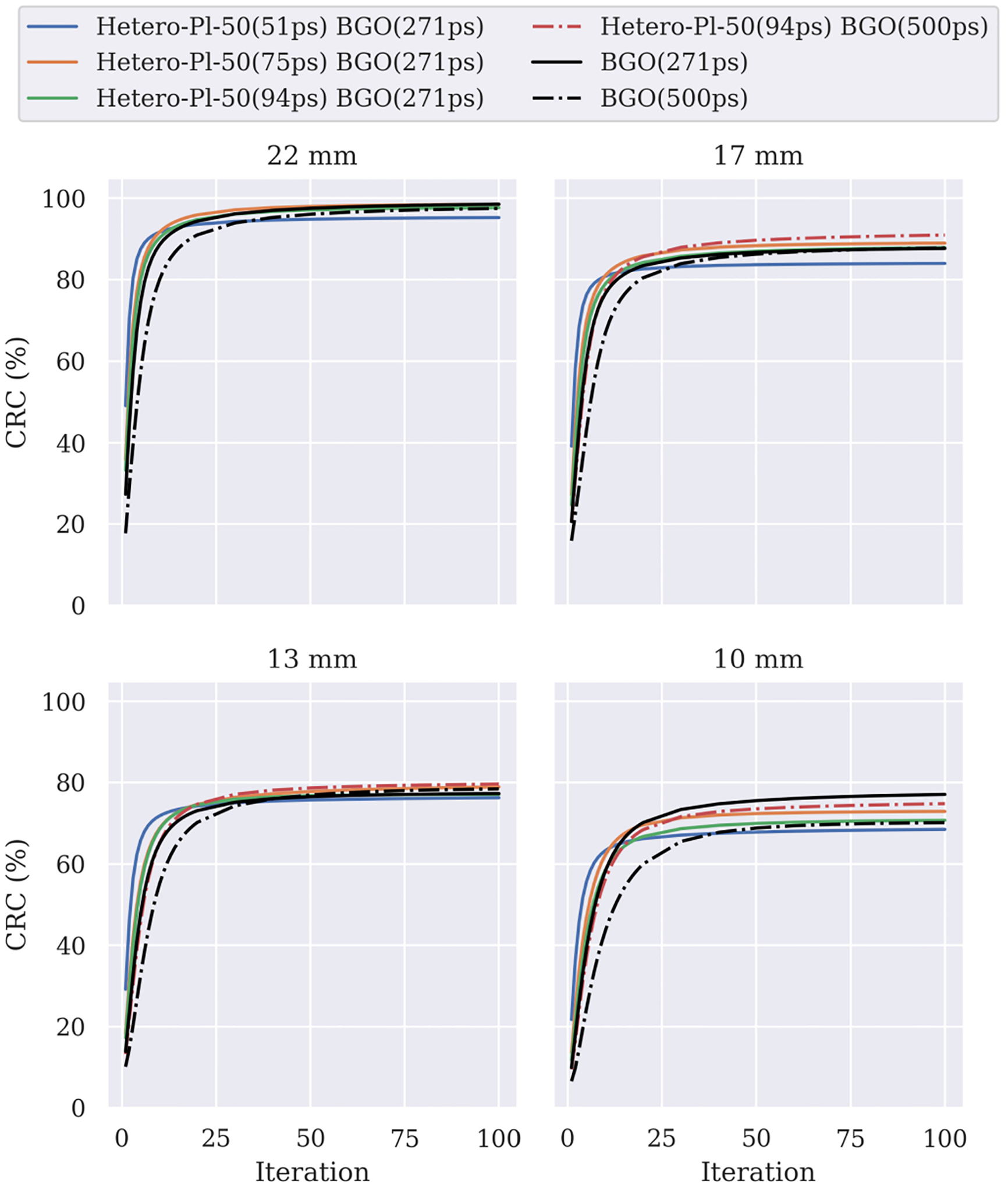
CRC of the four hot spheres of the NEMA IQ phantom, for the BGO (500 and 271 ps) and the Hetero-Pl-100 geometry, with *Pl* − *τ* 1 = 94 ps, *Pl* − *τ* 2 = 75 ps, and *Pl* − *τ* 3 = 51 ps, combined with the two BGO components. Similar curves were obtained for the Hetero-Pl-50 geometry.

In Fig. 7, we show the CRC for the BGO and Hetero-Pl-100 with the three time resolutions for plastic. We set the BGO timing resolution to 500 ps (system level) and 271 ps (laboratory level). For the 22, 17, and 13 mm spheres, the heterostructure has a slightly faster convergence than BGO (271 ps), which is more pronounced in the earlier <15 iterations. This improvement comes from the better CTR distribution. Also, as the BV suggests, we do not see a marked difference between 94 ps (Pl-*τ* 1) and 75 ps (Pl-*τ* 2).

The use of a worse BGO timing resolution (500 ps) did not affect much the convergence speed of the heterostructure. However, the impact on the bulk material is apparent. The above suggests that even a moderate amount of exploitation of fast Cherenkov events can have a noticeable positive impact on the contrast recovery.

Furthermore, we see that the 51-ps plastic loses contrast because, in this case, the shape of the timing spread heavily depends on the DOI, and thus the TOF modeling with a Gaussian function is not appropriate [32].

**Fig. 8.**
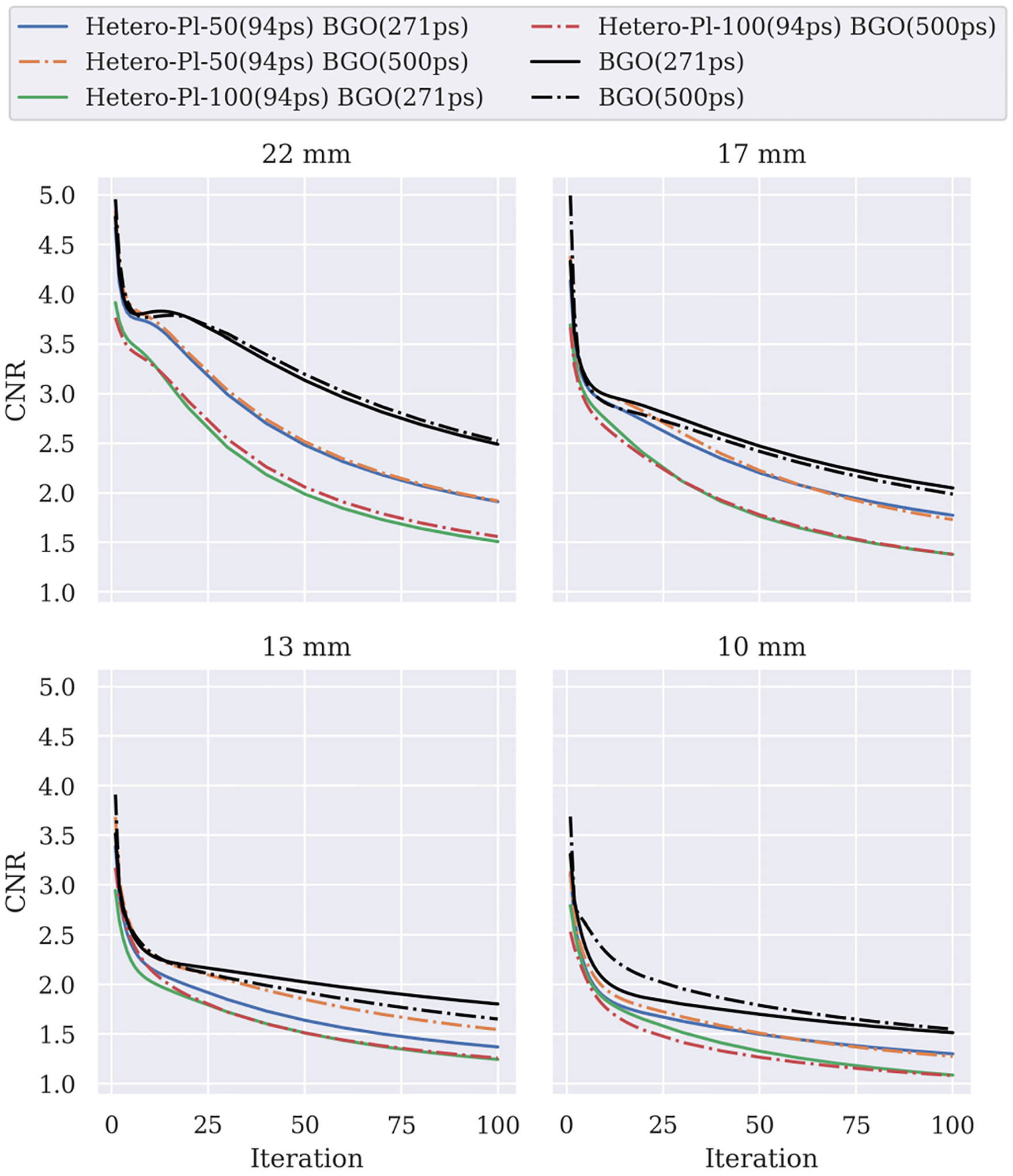
CNR ratio for BGO (500 and 271 ps), Hetero-Pl-50, and Hetero-Pl-100 configurations with 94-ps plastic time resolution (*Pl* − *τ* 2) combined with the two BGOs. Similar curves were obtained for *Pl* − *τ* 1 and *Pl* − *τ* 3.

In terms of CNR (Fig. 8), BGO has the best performance, followed by Hetero-Pl-50 and Hetero-Pl-100. The above was expected [41], as the three models produce images with very similar contrasts but have different detection efficiencies, which heavily influences the propagation of error in the CNR denominator. Specifically, concerning the two BGO models for the 13-mm sphere, we have to note that curves are driven by the SD in the volume of the sphere, which is 1.64 and 1.78 for the fast and slow BGO, respectively. The corresponding mean values in the sphere and SD in the background did not present any irregularities. This is a small difference, and at this point, we do not have evidence to suggest a systemic error; thus, we attribute it to the realistic statistics of the simulation.

The larger spheres show that the difference between the BGO with 500 and 271 ps resolution is negligible. As expected, the results on the smaller spheres are unclear due to the statistical uncertainty.

## IV. Discussion

This article investigated the potential impact of heterostructured scintillators in PET imaging. We demonstrated that heterostructures lead to a complex CTR distribution with slower and faster events. Compared to bulk BGO, heterostructures provide better contrast recovery in early iterations. However, we also saw a substantial loss in sensitivity and the effect of higher complexity in modeling the timing response of these detectors.

The CNR can best summarize the tradeoff between timing resolution and effective stopping power (sensitivity), which is a key takeaway message of this article. As shown in Fig. 8, in early iterations, the improvements in convergence keep the heterostructure geometry Hetero-Pl-50 with thin (50 *μ*m) plastic layers on par with the performance of the BGO-based scanner. However, after the 15th iteration, the BGO takes a clear lead due to the higher sensitivity. Unlike bulk detectors, heterostructures can be configured to optimize the said tradeoff, and the tools we present here can guide the design.

In the simulations, we considered two timing resolutions for BGO, one toward system level (500 ps) and one envisaging a BGO detector fully exploiting the detection of prompt Cherenkov photons (271 ps). When BGO is a heterostructure component, we did not observe a significant impact on either of the two figures of merit due to the difference in the input time resolutions. However, the effect on the bulk detectors is much more pronounced, with the contrast of BGO (500 ps) converging considerably slower than BGO (271 ps).

In the literature, the TOF SNR gain is described as proportional to sensitivity, more specifically to noise equivalent counts [42], and inversely proportional to the timing resolution 1/CTR [8], [43]. Thus, if the sensitivity is reduced by introducing the plastic layers to about half of BGO, we should aim to substantially improve the CTR to maintain the image’s noise properties. However, as discussed later, the DOI sets practical limitations on the potential CTR improvement.

The model for the calculation of the timing resolution based on the energy depositions shows that higher energy deposition in the fast plastic scintillator improves the CTR (Fig. 3). Moreover, the simulations showed that larger deposition could be achieved with thicker plastic layers, reducing the detector’s effective stopping power, as mentioned earlier.

In the two configurations, we saw that the fraction of events that deposit some energy in the plastic seems independent of the plastic’s thickness. The Hetero-Pl-50 offered the best compromise between photon detection efficiency and time resolution. Denser materials than plastic materials could be considered in future heterostructured detector designs with different geometry than stacking layers like fiber-based designs [24]. An alternative to plastic scintillators can be the nanocrystals [19], [35], [36]. Another approach could be to increase the pixel length [22]. However, it should be kept in mind that the latter approach introduces additional problems, such as poorer light transport and larger parallax errors, which affect temporal and spatial resolution, respectively.

We will add a few specific notes on the image reconstruction for heterostructures. As described earlier, the timing resolution depends on the energy deposition and sharing, leading to a complex TOF model for the scanner. However, here, the variety of timing resolutions obtained from the different combinations of detected events is much wider than in cases investigated previously [28], [43]. Efthimiou *et al*. [28], [43], studying Cherenkov-based detectors [44], [45], [46], [47], [48], [49], reported that the complexity of the reconstruction with multiple (25) kernels slowed down their convergence. In this work, with only three Gaussian kernels, the CRC converged slightly faster than the single and slower TOF kernel used for the BGO.

Furthermore, the time difference distribution’s shape depends on the average DOI of each material and the specific pathway in an event-by-event case [50]. We saw that the above led to a loss in CRC with plastic time resolution near the DOI of the material (51 ps) [34]. Our findings are in agreement with past studies [32], [51]. Also, several groups have proposed the time-walk correction or other methods [25], [52], [53], [54] to improve the shape of the distribution or account for it with bulk (pixelated or monolithic) crystals. However, heterostructures add another layer of complexity to this.

Therefore, while this study constitutes a good starting point to foresee the performances of heterostructured scintillators at a system level, further work is necessary to scale this design to a fully operative detector properly. First, we plan to simulate a single pixel, including the propagation of optical photons. The results may lead to the adjustment of our model and input parameters and the repetition of this simulation study. Second, the first step to scaling up our system experimentally will be to measure a matrix of 4 × 4 heterostructured pixels, which will also allow us to validate our model.

Another highlight of our reconstruction model is the possibility of investigating and optimizing how to make the best use of the events with very fast time resolution and to study the effects of the heterostructures on the positioning of the events and the spatial resolution.

## V. Conclusion

In this article, we presented the incorporation of a model to calculate the detector’s timing resolution based on the deposited energy of each gamma-ray in Monte Carlo simulations. This modification allowed us to simulate, for the first time, PET geometries based on heterostructured detectors.

Then, we advanced to reconstruct the simulated data using three TOF kernels and compared the IQ of the said PET detectors to BGO crystals with two timing resolutions. As we showed, the CTR depends on the energies deposited in the different materials of the heterostructured scintillator and the layer sizes. The images presented marginal improvements in contrast recovery and convergence of the algorithm due to the fraction of events with very fast timing resolution. This improvement was more pronounced with a larger difference between the two-time resolutions in the heterostructure, as demonstrated by using two different values for BGO. The Hetero-Pl-50 offered the best compromise between sensitivity and time resolution in the configurations studied here. However, introducing the low-density plastic layers strongly reduced the effective stopping power and thus the noise properties of the reconstructed image. A solution could be to replace the plastic with a denser scintillation material and/or to increase the pixel length.

The tools developed here can guide future heterostructure designs on the tradeoff between sensitivity and fast time resolution and evaluate their performance.

## Figures and Tables

**Table I:** Targeted CTR for the Simulation Output. Time Resolutions Were Corrected for DOI, and DTRs Were Used to Obtain the Target CTRS. Three Different Input Time Resolutions for Plastic (DTR_*Pl*@340keV_) and Two Different for BGO (DTR_BGO@511keV_) Were Considered

	CTR (ps)	CTR′ (ps) ^α^	DTR (ps) ^β^
BGO-*τ*1	271	266.2	188.2
BGO-*τ*2	500	497.4	351.7
Pl-*τ*1	94	79.0	55.9
Pl-*τ*2	75	55.0	38.9
Pl-*τ*3	51	0	0

α $CTR^{\prime }=\sqrt{CTR^{2}-DOI^{2}}$

β $DTR=CTR^{\prime }/\sqrt{2}$

**Table II:** Average CTR′ Per Group ($\bar{CTR^{\prime }}{}_{g}$), the Standard Deviation of CTR′ Per Group (*σ*_CTR′_), FHWM of the TOF Kernel Used in the Reconstruction (*fg*), and the (%) Proportion of Each Group for Both Heterostructures (Hetero-Pl-50 and Hetero-Pl-100), the Three Simulated Time Resolutions for Plastic and Two-Time Resolutions for BGO

Group	$\bar{CTR^{\prime }}{}_{g}(ps)$ ^α^	*σ*_*CTR*_^β^	*f*_*g*_ (ps) ^γ^	Proportion (%)
Hetero-Pl-50				
BGO-*τ*1-Pl- *τ* 1				
1	147.4	19.5	155.9	19.6
2	215.6	19.4	221.5	59.3
3	267.9	11.1	272.7	21.1
BGO-*τ*1-Pl-*τ*2				
1	130.8	26.7	140.4	33.3
2	212.6	17.6	218.7	51.1
3	270.5	10.9	275.3	15.6
BGO-*τ*1-P1-*τ*3				
1	1.2	1.1	51.0	41.7
2	193.1	9.9	199.7	45.8
3	273.2	9.9	277.9	12.6
BGO-*τ*2-Pl-*τ*1				
1	213.1	54.3	219.1	37.9
2	389.6	29.3	392.9	47.6
3	506.4	21.0	509.0	14.5
Hetero-Pl-100				
BGO-*τ*1-Pl-*τ*1				
1	139.2	23.3	148.2	35.3
2	216.6	17.7	222.6	50.8
3	272.9	11.3	277.6	14.0
BGO-*τ*1-Pl-*τ*2				
1	112.4	27.6	123.5	40.5
2	209.5	14.7	215.7	46.5
3	273.3	10.3	278.1	13.0
BGO-*τ*1-P1-*τ*3				
1	0.8	0.7	51.0	42.2
2	193.7	9.9	200.3	45.5
3	274.1	9.9	278.8	12.3
BGO-*τ*2-Pl-*τ*1				
1	173.0	49.9	180.4	41.6
2	382.7	24.3	386.1	45.7
3	511.1	19.4	513.6	12.7
BGO				
BGO-*τ*1				
1	271.2	9.5	275.9	100.0
BGO-*τ*2				
2	506.7	17.8	509.3	100.0

α Mean CTR′ of group (FWHM)

β Standard deviation of CTR′ in group

γ $f_{g}=\sqrt{{\bar{CTR^{\prime }}}_{\text{group}}^{2}+DOI^{2}}$

**Table III:** Proportion (%) of Shared Events for Two Heterostructure Configurations With 100 and 50 *μ*M Plastic

	Hetero-Pl-100	Hetero-Pl-50
Prompts	49.8 × 10^6^	82.9 × 10^6^
*E*_*Pl*_ > 0 keV (%)	63.5	43.1
*E*_*Pl*_ > 50 keV(%)	63.0	29.6

**Table IV:** Number of Coincidences Registered in the NEMA IQ Simulations

	Hetero-Pl-100	Hetero-Pl-50	BGO
Prompts	49.8 × 10^6^	82.9 × 10^6^	158.0 × 10^6^
Trues	27.9 × 10^6^	46.3 × 10^6^	88.4 × 10^6^
